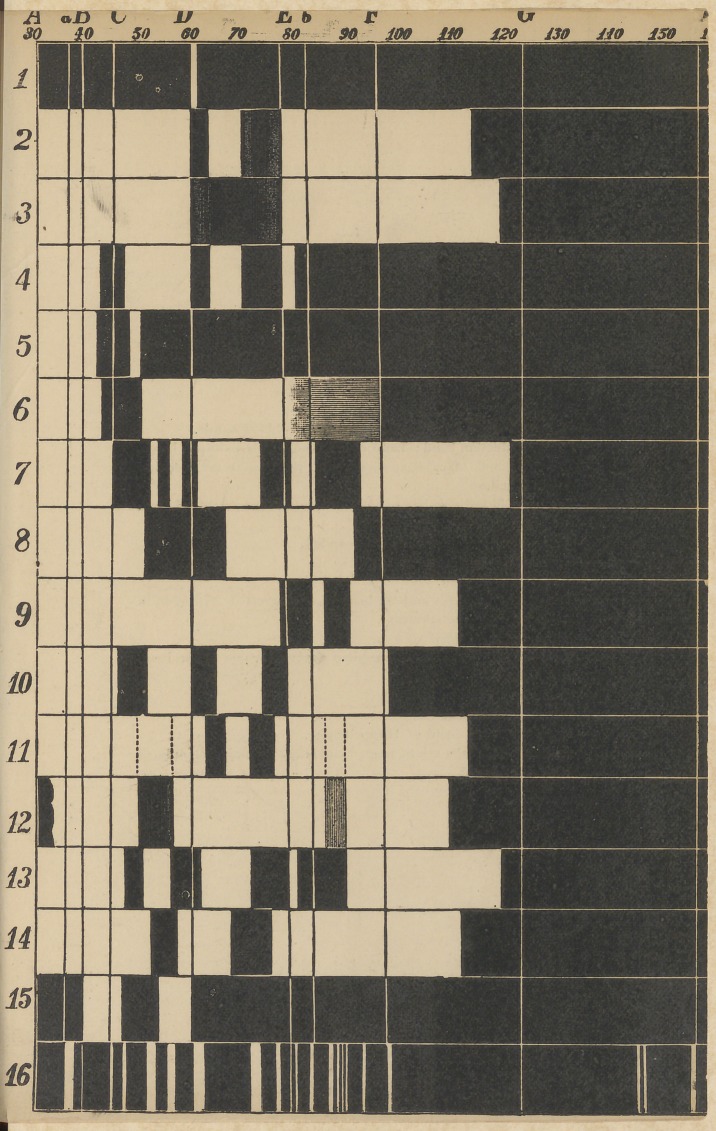# The Effects of Anæsthetics upon the Human System, as Evidenced by Spectroscopic Observations

**Published:** 1878-01

**Authors:** S. Waterman

**Affiliations:** New York


### THE DENTAL REGISTER.

VOL. XXXII.] JANUARY, 1878. No. 1.

### THE EFFECT OF ANÆSTHETICS UPON THE HUMAN SYSTEM, AS EVIDENCED BY SPECTROSCOPIC OBSERVATIONS.

*Read at a meeting of the American Dental Convention, the Southern Dental Association and the Dental Society of the State of Maryland, and District of Columbia, in joint session at Oakland, Maryland, August* 16, 1877.

BY DR. S. WATERMAN, OF NEW YORK.

*Mr. President, Officers and Members of the Convention, Ladies and Gentlemen:*

There is probably no difference of opinion in the profession, that a perfect anæsthetic, one possessing all the requisite properties to insure rapid action, complete safety, freedom from pernicious after effects, combined with cheapness and readiness in its preparation, would be one of the greatest boons to suffering humanity. And I am also certain that there exists no difference of opinion amongst us to-day, that we do not, as yet, possess this boon; that none of the anæsthetics known to us at present, possess all these priceless properties, and that, in dealing with these subtle agents, we are, indeed, passing the border land which separates life from death. I think it must be conceded that all anæsthetical agents now employed are more or less dangerous to health and life, and their employment is beset with more or less grave consequences. For estimating the effects of an anæsthetic upon the human system, its mode of action should be critically known before hand, not empirically only; we should be able to foretell what the action of a certain agent would be upon a given individual, by closely examining into the physical condition; and we should be fully able to appreciate the pathological states that forbid or modify its exhibition. To this end every rational practitioner is bound to understand the chemical composition of these wonderful agents, and, above all, what particular organ or fluid in the human economy is primarily effected by them; and also the precise manner of the changes which take place in the same. Those who imagine that all anæsthetics act upon the animal economy alike, and their peculiar mode of action is the same under all circumstances, have yet to learn that this is far from being the case; that on the contrary, various agents effect the system in quite different ways.

This knowledge, pregnant with the utmost importance, has become almost positive through the agency of the spectroscope. It has supplied the missing link to our chain of reasoning; the shadowy field of theories has been cleared up; the laws governing the relations of anæsthetics, in contact with the blood current, have been ascertained, and rational progress has been made to insure safe anæsthetics. I have abiding faith in the progress of chemical science, that it will finally point out an agent from the almost inexhaustable material at its command, that will satisfy all ends of surgical requirements; an anæsthetic that, whilst it will annihilate, temporarily all sensation, will leave consciousness and vitality intact. We are the more entitled to entertain this hope, as we are already acquainted with some agents, that, when locally employed, suspend the sensibility of the parts. Rhigolene is one of them, otherwise known as hydrate of pentyl, or amyl, a light fragrant fluid, the boiling point of which is 86° F. In the trimethylic ether we possess another remarkable agent of this class. Much of the knowledge we possess on these subjects has been supplied by the English savant, Dr. Richardson. I have clipped the following passage from his report of 1870: "In a previous report on amyline, I pointed out that its vapor, whilst it destroys sensation, does not destroy all conscious acts; and in my later observations on the action of methylic ether, C.7 H. 16, O3, the same facts have been more perfectly elicited. In several cases where I administered this ether, for removing pain in surgical operations, the patients, when quite insensible to pain, were so conscious that they were able to obey every request made of them, and in some instances were anxious to reason, stating that they knew what was going on, and arguing that they were not ready for the operation because they were sure they would feel pain. Nevertheless, in this state of mental activity, they were operated on, and afterwards, while remembering every incident, were firm in their assertion that they felt no pain whatever during the operation. One patient, who sat for the extraction of two teeth, selected the tooth to be first extracted, putting her finger on it, and afterwards re-arranging her position for the second removal. To the looker on, it seemed, in fact, as though no change in her life had occurred, yet she affirmed that she was sensible of no pain whatever; and several other less striking, but hardly less singular examples, came before me.

"We may, then, I think, fairly assume, that, in course of time, we shall discover, manageable and certain anæsthetic substances which will paralyze sensation only, leaving the muscular power unaltered, and the mental little disturbed; and we gather from this that either in the cerebral hemisphere there is some distinct and simple center of common sensation, which may be acted upon by certain agents without involving all the cerebral mass, or that the peripheral nervous matter may be influenced without involving those portions of the nervous system."

What Dr. Richardson here says is of the utmost importance on the subject before us. There may be cases where it may be useful, nay, necessary to suspend consciousness also, and we should be able to graduate our agent in a manner so as to push onward, to any desirable degree, without endangering the life of our patients.

Those who desire to learn more of this subject are referred to Dr. Richardson's most able and exhaustive report; and also to the highly interesting and admirable lecture by Prof. B. Silliman, Jr., of Yale, delivered to the medical class in Yale College, September 14th, 1871, and afterwards printed in pamphlet form, and in the *American Journal of Science and Art.*

I have already referred to the spectroscope, and told you that the spectral analytical test gives us most valuable information upon the subject before us. I propose now to make this assertion good. There may be many amongst my kind hearers that know all about the spectroscope, and the work it can do and has done for chemistry and celestial and terrestial physics Others, perhaps, may have given the subject less attention. For the benefit of all and in order to give a clear and satisfactory view, I shall speak as if this interesting subject was entirely new to you.

And in the first place, what is meant by the term "spectral analysis?"

It is a scientific process in which solar or artificial light is employed, in connection with a series of prisms, to analyze organic as well as inorganic substances. The instrument employed for this purpose is called a spectroscope, and when connected to a microscope we call it a micro-spectroscope. It consists of a number of prisms within telescopic tubes, and a slit arrangment so as to regulate the admission of light, and one or more collimator lenses to gather the rays and make them parallel. Through the movable slit the light enters and passes through the prism or prisms, and through one of the telescopic tubes the colored image or spectrum passes into the observer's eye, and is appreciated by the retina. This image may also be thrown upon a white screen, a method that I would gladly resort to had I possession of the necessary screen arrangements. You all know what happens when a ray of white light passes through a prism. It is decomposed into its ultimate constituent colored tints, forming a beautiful band called a spectrum. It contains all the colors of the rainbow, in regular succession of tints from red to orange, yellow, green, blue and violet. We witness, also, other interesting changes. When white light passes through a prism, the emerging rays are seen to have been bent out of their course.

They spread fan-like to the left and to the right. They are dispersed, and we call it the refraction of rays. The violet part of the spectrum is greatly more bent out of its course than the red part, which is less refrangible. This deflection and greater refrangibility of the violet rays, depends upon the constitution and nature of light itself, whose waves are propagated through space by a subtle fluid known as the "luminiferous ether, which fills the illimitable space and permeates every atom of matter. These etherial waves differ in length, the longest form the extreme red part of the visible spectrum, the shortest those of the extreme violet. According to Tyndal, the length of an etherial wave of the extreme red wouldrequire 36,918 placed end to end to cover one inch, whilst the extreme violet requires 64,631 to the inch." As the sun's light comes to us from a distance of 90,000,000 of miles, we can perceive the amazing number of waves and their inconceivable velocity, considering that these waves reach us in the short time of 8½ minutes. The number of ether impulses necessary to produce upon our retina the impression of red light, is, therefore, 451 billions per second, and in order to produce the impression of extreme violet, 789 billions are required. Impulses above, as well as below these numbers fail to make any impression upon our retina.

This, is indeed, a captivating chapter of physics, but I am admonished that my subject lies in a different direction, to which I am in duty bound to return.

When the light we employ for analytical purposes is artificial, say the flame of an oil or petroleum lamp, or the magnesium or electric arc light, we see the tints pass imperceptably, one into the other, and we have what is called an uninterrupted spectrum. When, however, sun-light is used, or the light from any planet that reflects the solar light, say the light of the moon, we find that the spectral band is traversed by thousands of fine lines, some darker and broader than others; such a spectrum is called an interrupted or solar spectrum.

The inquiry into the cause of these solar lines is full of interest, but I have neither time nor space to enter fully into its consideration.

These captivating features of spectral analysis are applicable to solar and celestial physics, but are not absolutely necessary to the intense, logical inquiry before us. The lines which traverse the solar spectrum are constant, and never change position. They have been mapped by Thalen and Angstrœm and Kirchhoff. Rutherford, of New York, has photographed a portion of them from the sun itself. Fraunhofer employed the most prominent of these lines for purposes of measurement as far back as 1814. He selected 9 lines in various parts of the spectrum and named them A, B, C, D, E, F, G, H and b, and these lines are known the world over as Fraunhofer's lines. You will understand by and by how useful these lines are in spectral analysis. These 9 lines, and in fact every one of the thousands of lines that traverse the spectrum, represent some terrestrial substance in a vaporous condition in the sun; and we learn from those hieroglyphic lines, that the sun, the stars, the comets and the nebulæ, the aurora borealis and the zodiacal light, which, according to the latest view, encircle our earth as Saturn is encircled by a triple set of rings, that in short, all celestial bodies, without exception, contain substances or elements which we meet on our earth, thus bearing witness to the unity of the Universe.

The D, line is produced by burning sodium; the lines C, F, and G, are peculiar to burning hydrogen gas; the E, line is one of the most prominent iron lines; the line B, is produced by the vapors of magnesium, and the H, line is characteristic of volatalized calcium. In our inquiries these lines serve us as landmarks to register the position of bright lines and absorption bands. Scales, graduated into tens and hundreds of degrees, are also employed and placed above the spectra dividing the color regions.

The process of making an analysis by means of the spectroscope is simple indeed. Bodies to be examined are either solids, liquids or gases. The solids are volatilized by means of heat. To this end we employ a Bunsen's burner, the electric arc, or the compound oxygen flame.

Fluids are placed before the slit of the spectroscope in suitable glass vessels, with plane parallel walls. When the rays of light pass through colored solutions, ere they impinge upon the prism, various tints are absorbed. We observe a variety of dark bands, varying in shades, in numbers, and in position in the spectral regions. There are no two substances, at present known, that give absolutely the same bands.

Gases are examined by means of tubes devised by Pflucker & Geisler and known as Geisler's tubes. They are made of various sizes and shapes, some quite fanciful. They consists of their thermometer, tubes with a bulb at each extremity, into which electrodes of platinum or aluminum are soldered. Electrodes of other metals would oxidize in the extreme heat generated. The tubes are filled with the gas we wish to experiment upon. The air pump is then applied until the 1-600 or 1-700 part of the ordinary atmospheric pressure is left. Then we pass on electric spark through the attenuated gas, which in this condition, no longer resists the passage of the spark; intense heat is generated, and brilliant and beautiful lights emitted, of various colors, changing of course with the different gases employed.

Being able then to master the solids, fluids, and gases, no known substance can escape the analytical power of the spectroscope. Every known substance modifies the spectrum in a manner specific or peculiar to itself. Some substances give only bright lines, for example the glowing gases, others give dark lines and absorption bands. Some absorb all the colors of the spectrum with the exception of a single bright line. Observe, in the subjoined diagram, the spectra of sodium and thallium. Others give a spectrum of many bright lines; compare the spectra of barium, caesium, and rubidium.

It does seem at first sight, that the immense variety of lines and bands would lead us into inextricable confusion. A little practice will dispel this illusion however. We soon become familiar with these landmarks. The variety of the spectra, the relative position of bands and lines, their peculiar forms and outlines, difference in brightness, depth of shading of bands and their number in each instance, are characteristic enough to insure ready recognition, even by persons not accustomed to work with the spectroscope.

DELICACY OF THE SPECTRUM OR PRISMATIC TEST.

Let me say a few words regarding the extraordinary delicacy of the spectrum test, which far surpasses every other test known to us. The following example is supplied by Dr. Schöllin: "Let us divide one pound of common table salt, the sodium chloride into 500,000 equal parts. One of these minute dust particles is called a millogram. The experienced chemist is able to weigh such a minute particle only with the most delicate scales and with extraordinary care and acquired dexterity, but with this performance he has arrived at the limit of possibilities. And now ask the chemist to divide this millogram into further 3,000,000 equal parts, and he will shrink appalled from the performance of this impossible task. The human mind cannot conceive of an object so exceedingly minute. Yet we can demonstrate the presence of such an infinitesimal quantity of sodium chloride by the spectral test. You know that this salt is ever present in nature in extremely fine division. Its never failing source is the sea; fine particles of it are supplied to the air by the action of winds and storms and by the slower processes of evaporation, thus supplying one of the most absolutely necessary elements to life in its manifold forms and conditions, and furnishing one of the most powerful antiseptics, whereby contamination of air, earth and water is prevented.

The dusting, or slapping together of dusty books in the remotest corner of this hall will immediately produce a yellow flash in a burning candle or gas flame at this end, which when examined with the spectroscope will show most distinctly the yellow line in D, which, as you have already been informed is the sodium line.

There is another very peculiar and highly useful characteristic of the prismatic test, to which I desire to direct your attention. You can examine a number of spectra at one and the same time, that is, you can analyze a number of substances at the same time. Take, for instance, the ash obtained from the incineration of human, or animal tissues. The hydro-chlorate solution of this ash gives a splendid spectrum, the field of which shows many red, yellow, green and blue lines in various regions of a dark spectral ground. (*)

By careful comparison we find that these lines belong to six metals, to-wit: potassium, sodium, lithium, rubidium, cæsium, and calcium. We can give another striking example. The ashed end of a cigar, moistened with hydro-chloric acid and held in the flame of a Bunsen's burner, yields the lines of sodium, potassium, lithium, cæsium, rubidium, calcium.—(*Thudicum's report to the privy council*, 1876.)

Four new metals were discovered by means of the spectroscope, of very great interest to science, which would otherwise, most probably, have never been known to us. Bunsen and Kirchhoff discovered, in the waters of Durckheim, cæsium and rubidium; for, boiling down forty tuns of its mineral waters, they found 200 grains of the mixed salts of the above metals, and by the marvelous analytical powers of the spectroscope, indentified these substances. (1860.) Since then these metals have been found in many other localities, especially rubidium, to which many of the most celebrated springs in Europe owe part of their curative powers. Thallium, a most important metal, was discovered by Crook, in 1861, in some of the iron pyrites and in a seleniferous deposit from a sulphuric acid factory, at Telkerode, in the Hartz. (*Roscoe.*) Reich and Richter discovered, in the same way, the red metal indium, (1864) on account of its spectrum, two indigo-blue lines.

You may rightly conjecture that an instrument possessing such wonderful analytical powers, must have found application in manufactures, arts and sciences; its influence upon celestial chemistry is simply stupendous; it has completely revolutionized our views in this direction. A comparison of the dark lines of the sun, and its planets, with those of Sirius, and other fixed stars, shows us, that the same substances known to us are present in all these. Differently arranged as those lines are in different stars, many of them are sufficiently coincident to establish their indentity. When we come to examine the irresolvable nebulous mass, we obtain no longer dark lines, but bright lines only; and we learn thereby, that these bodies consist of burning gases,

*See diagram.

principally hydrogen, which is also so abundant in the sun, when, during the fire storms raging there, it is carried up with explosive force, many hundred thousand miles, in the shape of fiery columns.

As we come down to the still lower grade of cosmic evolution, to the nebulous mass, even these bright lines diminish in number, until but a few of them remain visible. One line in F. seems to be always present, the line is nearly coincident with the hydrogen line; another seems to indicate the presence of nitrogen, and still another has not as yet been identified.

In the comets all the bright lines have disappeared. Faintly illuminated spots mark the place where, in ages to come, bright lines will appear. These spots correspond to the spectrum of carbon, and one of these comet worlds may weigh only a few hundred pounds, and may not contain more solid matter than can be stowed in one's hat.

In time to come, when the comet's cosmic dust will have been contracted and condensed, and heat and light will have been evolved, bright lines will mark its progress, and in due time again, as condensation progresses, dark lines such as we now observe in the spectrum of our own sun, in the spectrum of Sirius and that of a host of other stars, will become visible, and in many, many million years, perhaps, when our sun system shall have become old and frigid, and its light and heat shall have been dimmed and exhausted, and life has become extinct in consequence thereof, the host of nebulous bodies, now in progress of being born, will assume all the brightness of our present sun, and light up the chaos consequent upon the disappearance of our present sun-system.

I have scarcely any time left to point out the use of the spectroscope in the arts and manufacture. You must be satisfied with one example. You have heard of Bessemer steel. Steel differs from iron in containing less carbon. In the Bessemer process, carbon and silicon are burned out by oxygen, contained in a blast of atmospheric air, which is thrown through the mass of molten iron.

Formerly the manufacture of Bessemer steel was surrounded by great difficulty, for it is necessary to recognize the exact moment when all carbon is burned out of the iron, ~~or the cast is lost.~~ When this exact moment has arrived, the operation must be stopped instantly; ten seconds more or less will destroy the entire cast. The spectroscope shows this exact moment when the process is finished, and makes the manufacture of Bessemer steel at once an easy and successful task.

I think I have touched, like Ithuriel's spear, lightly upon the most salient, technical points necessary, so that you may understand, in how varied a manner, the spectroscope may be utilized.

We come now to its application in medicine, and I claim that its usefulness and importance here is second to none I have already mentioned.

But to understand how it can show us the effect of anæsthetics upon the human system, we must be familiar with the constitution of the blood, and learn the optic relations of this vital fluid to the spectroscope, in health and disease.

The optic phenomena of blood were not known prior to 1864, when almost simultaneously, Hoppe Seyler, in Tubingen, Germany, and Dr. G. G. Stokes, in England, investigated this subject. Stokes pointed out the fact that blood causes a peculiarly strong absorption of light in the yellow and green part of the spectrum. In order to observe this absorption well, the blood should be properly diluted, for in its concentrated state, the absorption bands between D and E, cannot be observed at all.

These two bands are beautifully dark or black, the first which is narrow, more so than the second, which is broader; both are known as the spectral bands of oxidized blood. But blood can exist in a double state of oxidation; that is, it may also exist in a state of complete de-oxidation. The oxidized blood corresponds to the arterial; the de-oxidized to the venous blood. When the blood is de-oxidized, the two bands disappear, and are replaced by one dark, broad band, known as Stokes' reduction band. This black band filling the space between D and E, appears whenever the blood is deprived of its oxygen, which it loosely binds.

This de-oxidation may be effected by mechanical as well as chemical means. The blood is then called reduced or de-oxidized blood.

We may deprive blood of its oxygen mechanically by means of an air pump favored by heat. Chemically, blood may be deprived of its oxygen by substances which have an energetic affinity for oxygen and absorb it whenever they find it. Tin ammonium sulphide and others. Blood thus reduced, or de-oxidized, may be rapidly re-oxidized by shaking up the solution with atmospheric air.

The spectroscope is not idle during these changes; the reduction invariably causes the appearance of Stokes' reduction band; the re-oxidation causes the re-appearance of the two beautiful bands between D and E. The same changes take place in the living economy.

In the mean time the discovery was made, that blood contained a crystallizable material called hæmato-crystalline, also hæmo-globulin and cruorine. The great practical importance of this substance must be my apology for entering more minutely into its consideration.

Hæmato-crystalline is the agent through which oxygen is abstracted from the air, and loosely bound in the circulation. Take this crystallizable matter out of the blood and the residue, consisting of albumen, globuline, protagon, cholesterine, sulphur, iron, and some salts, will be quite unable to effect this attraction of oxygen. Hæmato-crystalline saturates itself in the lungs with oxygen, it carries its precious burden into the sanguineous circulation, and sustains there the energies of respiration, oxidation, and oxygenation. In its course it gives up its oxygen, thus absorbed to all oxidizable tissues with which it comes in contact, and in exchange unites with carbonic acid, which through the venous circulation is brought back to the lungs for elimination, by a process not yet fully understood.—(*Hoppe Seyler.*)

It must be evident to you, that so long as the hæmato-crystalline of the blood remains intact, the same in quantity as in quality, the supply of oxygen to the living economy is subject to relatively unimportant oscillations, and that with the increase or decrease of this substance, or with any change in its integrity, rises or falls the vital capacity of an individual's life.

That it is beyond any question, the hæmato-crystalline, and not any other substance of the blood which enters into and sustains the vital exchanges between the oxygen and carbonic acid, is fully proved by spectral observation. Hæmato-cryst. artificially prepared and in solution, is able to absorb oxygen as well as carbonic acid with great rapidity. It presents two states of oxidation, the arterial and venous. It can be oxidized and de-oxidized at pleasure; it presents the same absorption bands as blood; it can be reduced, and when shaken up with air, can be readily re-oxidized. It presents the same optical changes when reduced or altered. It enters the same combinations with irrespirable gases, in short all and every chemical and optical appearance which blood presents when acted upon by chemical as well as mechanical agencies, are also observed when these agents act upon a solution of hæmato-crystalline.

Highly interesting experiments, instituted by Pfluger upon dogs, have given us information of the rapidity with which oxygen is used up in the living animal economy. This physicist, forced these animals to inhale nitrogen, which you know does not support respiration. In thirty seconds the highest point of dyspnœa was reached.

At this point some blood was abstracted, under the necessary precautions, and then tested for oxygen. It was found that its oxygen was reduced to a minimum, being 1 to 2 per cent. whilst the blood abstracted from the same animal immediately before it was forced to inhale the nitrogen, contained 18.6 per cent. of the vital gas.

As soon as the animals were permitted again to inhale the pure air the dyspnœa disappeared and they seemed as well as ever.

You perceive, gentlemen, I have guided you gradually up the hill from which your views will become clearer and fuller. If you will but grasp these facts, presented to you, you will have no difficulty in mastering what follows.

Were it possible to observe in the spectroscope the changes taking place in the blood of such a suffocating dog, we would witness a rapid fading away of the two oxygen bands between

D and E, and towards the end of the catastrophe one dark band would take their place, the reduced or de-oxidized band of Stokes'; and by the time this band had obtained its full extension and its full depth of shading, poor dog Tray will have gone to his eternal hunting ground.

And here comes in the first great lesson in the administration of anæsthetics: "That suffocation will rapidly ensue where anæsthetics are used, which cannot sustain respiration, or, which is still worse, abstract what supply of oxygen the blood has stored up, unless a sufficient supply of atmospheric air is permitted to be inhaled to sustain life at the same time."

The great rapidity with which the dogs, experimented upon, recovered, shows us that the blood itself had not been fatally injured or altered. In taking the blood drawn at the height of dyspnœa, and shaking it up with air, the spectroscope would have promptly informed us of the reappearance of the two oxgyen blood bands, in full depth of shading.

To Pfluger's experiments we owe another series of important facts. The amount of oxygen contained in the animal and human blood, 16.9 per cent. Blood-serum contains less than one per cent. The more compact and normal the blood, the more numerous the blood corpuscles are, the greater is the per centage of hæmato-crystalline, the greater is also its capacity to absorb oxygen; the poorer the blood, the smaller is its amount. One grain of hæmato-crystalline is able to bind 1.27 c. c. of oxygen.

When we examine spectroscopically the blood of chlorotic persons, or that of persons who have sustained severe hemorrhages, or who suffer from pernicious ænemia, bright's disease, fatty degeneration of the heart, or the blood of persons in whom disease has reduced the crystallizable coloring pigment of the blood, as is the case after cholera, typhoid and other diseases, we find the bands paler, and know at once that the normal amount of oxygen is wanting in such individuals.

Here then comes in our second great lesson. In all cases, due inquiry should be made into the history of the person who is to be placed under the influence of anæsthetics, and if it is found that any of the diseases enumerated above has been present, and that the hæmato-crystalline has been reduced by disintegration and retrogressive processes, leaving your patient with pallid countenance and defective heart's action, be on your guard, for what remains, of the vitalized blood, may not be able to resist the effect which your anæsthetic is apt to produce, because in these conditions every anæsthetic agent is dangerous.

Let me briefly make you acquainted with agents which permanently alter the blood. All acids as well as all alkalies are such agents. With these changes we witness corresponding changes in the spectrum, quite definite and characteristic. Here you see on the diagram various spectra resulting; Hæmatic and Cruentine, Hæmatoidin, Hænim. I have no time to dwell upon their great importance in spectral analytical investigation, but will refer to them by and by.

The blood crystals of which we have so often spoken are not found in crystalline form in the blood. They are present there in solution joined to an alkali, probably to potassa carbonate, forming hæmo-globulate of potassa. (Preyer,) Hæmato-crystalline is a weak acid. It can be produced pure, but its preparation is difficult. It crystallizes in rhombic prisms of great beauty and bright red color.

AFFINITY FOR IRRESPIRABLE GASES.

Wonderful as are the functions of this crystalline material, it possesses qualities whereby destruction to life is invited and facilitated. They have an exceedingly energetic affinity for irrespirable and poisonous gases, with some of which they enter into close and inseparable combinations, thereby sacrificing their own integrity and life-supporting power for ever. Some of these irrespirable gases simply disables the hæmato-cryst, of the blood to absorb oxygen; others consume all the oxygen of the blood to satisfy their own keen affinity for this gas; others cause a cleavage, or true chemolysis of the hæmato-cryst, combining with its alkaline base, setting free the crystallizable material, whilst still others cause several of these effects to take place at one and the same time.

When a cleavage of the blood material has taken place, the disintegrated elements become foreign bodies and must be elliminated and carried from the system.

Preyers experiments upon the dogs, reminds you how rapidly the oxygen is consumed in the animal economy and how necessary it is to supply the defect in our equally speedy way; and you can understand how rapidly a fatal result must insue from the action of anæsthetics which, cannot supply the defect, and which in addition greedily appropriate the oxygen which the blood may have stored up and still further destroy the integrity of the hæmato-cryst. in a manner so as to paralyse its vital functions. Some of these combinations can be obtained in crystalline form. We can thus produce the prussic acid, the carbonic oxide, and the nitric oxide hæmato-crystalline. The combinations are far stable than the normal oxyhæmato-crystals are.

When blood has once entered into a permanent crystalline union with nitrous oxide gas, we know as yet, of no chemical process to restore the resulting nitrous-oxide hæmato-crystalline to its normal condition. No electric current posesses the power to restore the primitive integrity of the blood when once brought into this fixed condition.

Recently Donders and Young have demonstrated and Podalinsky Julenburg, have corroborated that the hæmato-crystalline may be released from the deadly grasp of carbonic oxide by means of carbonic acid, hydrogen and oxygen, being persistently passed through a solution of carbonic oxide hæmato-cryst., so that the blood band of stokes and finally the two bands of oxy-hæmato-cryst. may be reproduced. Whether such a process would succeed, in case of blood saturated with nitrous oxide, whose grasp upon the oxygen of the blood is far more tenacious, is a question which cannot be answered at present.

NITROUS OXIDE GAS.

Let us begin with this gas, the so called laughing gas, the one so extensively used by surgeons and dentists, and by many considered a serviceable and harmless agent.

It has been demonstrated by Herrman and verified by Hoppe Seyler, Gourp Besonez and W. Preyer, that Nitrous oxide gas possesses a very keen affinity for oxidized blood as well as for artificial oxy-hæmato crystalline in solution. The affinity is so strong that when a current of this gas is passed through a solution saturated with carbonic oxide, hæmato-crystalline, the carbonic oxide is driven out by the nitrous oxide, which takes its place volume for volume.

When a current of nitrous oxide gas is forced through a slightly alkline solution of hæmato-crystalline, the solution looses its dechroism and assumes a slight cormaisin red color. When the solution is placed before the spectroscope we observe that in proportion that the gas exerts its influence, the two bands between D, and E, fade away and disappear finally altogether, and there is a moment, says Preyer, "when the spectrum is continuous."

The disappearence of these blood bands means here, as it means in other instances, disappearance of oxygen from the blood, or complete deoxidation, and unless a fresh supply is speedily furnished, sucffoation must ensue.

As the action of nitrous oxid gas upon the blood solution continues, soon after the fading away of the two bands, two new bands appear, resembling the oxy-blood bands, but differing from them in position and depth of shading; they are paler and more blurred in outlines.

Please remember, in this connection, what I said to you about Stokes' reduction band. I then told you that when blood is simply deprived of its oxygen, the blood reduction band would follow the disappearance of the two oxidized blood bands; and that then, the simple contact of atmospheric air with such de oxidized blood solution would suffice to cause the re-appearance of the two oxygen blood bands.

But we see here, that instead of Stokes' band, two entirely new bands have made their appearance; and when such blood, saturated with the nitrous oxid, is then submitted to the action of reducing agents, the broad band of Stokes, the reduction band, can no longer be produced at all, proving that a more permanent change has taken place in the vital chemistry of the blood.

When a current of nitrous oxide gas is passed through a blood solution not made previously alkaline, still further changes take place. Here a portion of the nitrous oxide gas rapidly oxidizes, at the expense of the oxygen of the blood, and forms hyponitric acid. Preyer holds that this hyponitric unites with the hæmatocryst of the blood in its nascent state. Like all acids, it alters and suspends the coagulability of the blood, and initiates other important chemical and optical changes.

This event is marked by the appearance of an absorption in red to the left of D, from the 53° on Preyer's scale towards D, and another one between b and E. I look upon the appearance of this absorption in red as an indication that the union of the hyponitric acid has formed and has united with the blood. We already learned that all acids, cyanic acid excepted, cause a decomposition of the blood, and its product is hæmatine.

Now let us logically apply all these ascertained facts to our case in hand, in order to learn how this gas produces its effects upon the economy.

It deprives the blood of its oxygen, and ~~by entering~~ into a close combination with its crystallizable material; so bound, it disables this latter to absorb oxygen from the air, or to supply it to the oxidizable tissues of the economy.

In Preyer's experiments we have seen that the dogs, when permitted to inhale oxygen at the highest stage of the disponea, they became rapidly as well as ever. Not so after the inhalation of nitrous oxide gas.

A certain effect upon the blood has taken place; often un important and transient; at other times more permanent and grave, sufficient at times, to endanger life itself.

We have also seen that under favorable conditions, hyponitric acid is formed, which causes a decomposition of thehæmato-crystalline into hæmatine a substance which is not capable of sustaining life.

Thus we are forced to acknowledge that the application of this gas is far from being safe and harmless; that on the contrary it is pregnant with grave consequences.

"These facts," says a writer in Braithwaite's Reprospect, No. 67, July 1873, "ruthlessly destroy the infatuation, that the inhalation of nitrous oxide gas is a harmless process, a process which any man, educated or not educated, may carry on without danger of destroying life. The recent death which has occurred at Exter on the afternoon of January 22nd, of this year, furnishes a lesson not to be forgotten. The gas was administered by Dr. F. F. Mason, for the purpose of the painless extraction of a large upper, molar tooth. The lady, Miss Wyndham, was about 38 years of age, in good health. Her physician, Dr. Pattison, was present. Gas from the same source had been administered to other patients so that its quality could not be impugned. She took the gas in the usual way, without any symptoms to excite uneasiness. At the proper degree of insensibility the gas was stopped and the tooth extracted. It was not until after the operation was completed that anything unusual happened; her face suddenly became livid, and the features began to swell, and she seemed to be quite unconscious. She breathed two or three times and in a few moments her pulse ceased to beat. All attempts to restore her were fruitless."

"There was no obstruction to the air passages, and the tongue was protruded while she still respired."

The writer continues; "From no agent have there been so many hairbreadth escapes from death as from this gas, and probably of late some persons every day have been brought within the minutest line of danger to which Miss Wyndham succumbed.

We learn the most important lesson that we have a great deal to learn before we shall have perfected anæsthetic agents, toward such learning the re-introduction of nitrous oxide gas has been a serious check.

"Nitrous oxide gas is indeed not a true anæsthetic at all. A true anæsthetic is an agent that suspends common sensibility without, by any necessity, interfering with those organic processes on the continuance of which life depends. Nitrous oxide gas acts by suspending one of the most important of the organic processes, that of respiration. The insensibility produced by this gas, is afforded during an interval of partial death. This interval, doubtful, transient, dangerous, may allow an operator time for a short operation, and suspending the inhalation the function may return; but that it may never return the above case furnishes a lamentable proof."

This was written in 1873, and many other cases of death from this gas, are since recorded. Two years previous to this, in

1871, my warnings had reached England, and had in part been published there in the *Medical Times* and *Gazette*, London, January 13th, 1872. It was a lecture delivered before the New York Academy of Medicine, in 1871. Touching nitrous oxide gas I made the following remarks: "No intelligent observer, who ever witnessed the ghastly, cyanosed appearance of persons who have inhaled this gas until its anæsthetic effects are produced, will deny that the ensemble of symptoms betokens a powerful influence upon the blood mass which continues for many hours and days. I have seen some cases where the inhalation of this gas was followed by pulmonary and cardial disease and death. The profession at large, may yet learn to modify its opinion, regarding its freedom from danger after its application."

You may remind me that in a dental institution in New York, we are shown a gigantic roll, containing the names of many thousands who have inhaled this gas there, so far, without any direct fatal effect.

But how is it with after effects? The institution referred to keeps no record of what becomes of its patients afterwards. One of my fatal cases—dying from after effects—had inhaled the gas there three or four months previous. She was a perfectly healthy woman before the inhalation, and her disease began right after it. Other well authenticated cases are not wanting to prove, that nervous disorders, of many kinds, and a train of organic diseases, follows its exhibition. Dr. F. R. Thomas, in his treatise on "Nitrous Oxide gas," says: "It resembles strongly in its effect an attack of Congestive Apoplexy. Many are deprived of sleep long afterwards, and complain of unremitting headaches; nor are instances rare, where, after its use, vertigo, syncope, melancholy, insomnia, convulsions, hesteria, and irregular heart's action, could be attributed directly to its use." (Dr. Geo. J, Ziegler's researches on nitrous oxide.)

Having fully pointed out to you the manner in which nitrous oxide gas effects the blood, it must serve you as a type for all those agents which deprive the blood of its oxygen, and form stable crystalline compounds with the hæmato-crystalline ~~of the blood,~~ whereby ~~their~~ life function is gravely impaired, and under certain conditions forever lost.

In case of accident with nitrous oxide, our indications are confined to narrow limits. We must try to economise the still intact blood corpuscles, and by transfusion, and especially by artificial respiration, to favor a full and long supply of oxygen to sustain the little flame of light. Electricity may be used to keep up the muscular action of the heart and lungs. We may thus succeed to organize the accumulated nitrous oxide, and to eliminate it from the system. Podowsky has thus succeeded in some almost hopeless cases of poisoning with carbonic oxide, and the proceedure seems to me well adapted also in cases of poisoning with nitrous oxide gas.

The next class effects the blood by causing a mechanical breaking up of the blood corpuscles, or a true chemolysis of blood element. This class, says Hoppe Seyler, does not effect the function of the hæmato-crystalline, but prevents oxygenation and oxidation in the tissues.

### CHLOROFORM.

The introduction of chloroform into the blood current is noted for its energy and corresponding danger. Experiments, made with this agent upon the blood shows, that the hæmato-crystalline is precipitated from its solution, a gelatinous is formed leaving a ghost of a stroma in the shape of empty cell walls.

Preyer says: "That the spectrum of the precipitated hæmato-crystalline is normal, and the two bands between D and E are clearly seen. The force of chloroform is therefore not spent upon the hæmato-crystalline, and the mischief done must be sought in the results of a cleavage in which the hæmato-crystalline is forcibly expelled from, and exudes from the blood corpuscle. It is probable, also, that it is thus forcibly separated from the potassa carbonate. In a fatal case of chloroform inhalation, reported in the *New York Medical Record*, Sept. 1st, 1877, post-mortem examination revealed, that the immediate cause of death was found to be effusion of blood upon the brain. It will be seen that the foregoing view tallies with this pathological condition. Chloroform forms no combination with the blood in the manner nitrous oxide acid and other chemical agents of like nature do.

Chloroform possesses another characteristic that adds to its fatal influence when inhaled; it is its high boiling point, requiring a great amount of vital force, a great amount of oxygen to organize it and to eliminate it from the economy. It is a well ascertained fact, that in all anæsthetic agents the boiling point of each is of the highest consequence. The higher the boiling point the greater the probability of danger. Prof. Silliman says: "The main disadvantage of chloroform is its high boiling point, requiring a great amount of vital force to eliminate it from the body, so that it is probably never eliminated entirely by the lungs, but only with the aid of all excreting organs, any deficiency or derangement of which may consequently lead to such suppression of elimination, that the nervous system may be overwhelmed with consequent arrest of their activity." (Silliman's lecture, 1871.)

It is but fair to state, that here, also, is a great deal to learn of the mode in which chloroform spends its force in the living economy. The warning given when I spoke of nitrous oxide gas, regarding the danger to give this agent to debilitated persons, or to those laboring under organic disease, and in impoverished conditions of the blood, must find a still more grave consideration here. How the extravasated hæmato-crystalline is carried out of the system is a matter of surmise. I found very frequently sugar in the urine after the inhalation of chloroform.

Prof. Silliman thinks the best treatment in impending death, from chloroform, is the introduction of air into the lungs by artificial respiration—heated to 130° F.—by means of bellows.

ETHER.

The action of ether is somewhat different. You remember, that in speaking of the blood corpuscles I mentioned protagon and cholesterine among its constituents. Albumen, protagon and cholesterine form the medulary part of the nerves, and their great importance to the animal economy is conceded by all hands. They are found stored up in animals as well as plants, and support all vital processes of germination and growth.

Formerly indeed their presence was not considered of very great importance. It was thought these substances had only a subordinate function to perform. We know now that they furnish elements for the brain, and are ever present in the primitive vital elements of the seminal fluids, the yolk of eggs and in the red as well as white blood cells. Ether dissolves these important factors out of the blood, as well as out of vegetable seeds. Such as peas, beans and lentils; it breaks up the determined constitution of the blood cells, and thus affects directly the hæmato-crystalline by severing its association with potassa carbonate.

When ether is shaken up with fresh blood a gelatinous mass is formed; the bright cherry red is changed into a muddy, brown colored pigment. When this change is observed by means of gas chamber, such as used by Stricker and Lancaster, and the spectroscope, we find that in addition to the oxyhæmato-crystalline band between D and E, there also appears a third band near C in the red part of the spectrum. (See Preyer, die blutcrystalle, p. 146.) The band in red always denotes that grave changes have befallen the blood, and Hoppe Seyler thinks it is due to the formation of methæmoglobin.

Fortunately for suffering humanity the boiling point of ether is far below that of chloroform and less oxygen is needed for its elimination from the system. Yet it has also its death roll like chloroform and other agents, less appalling it is true, but still all caution is necessary and eternal watchfulness and care.

And now the end has come, undoubtedly to your great comfort and relief. I thank you for your undivided attention during the delivery of my remarks. I hope they may stimulate thought and original investigation, and give encouragement to unceasing efforts, until the great boon to humanity "a perfect anæsthetic" is found.

---

### GALVANIC ACTION WITHIN THE MOUTH. ITS RELATION TO DENTAL SURGERY.

BY GEO. WATT.

*Written for the Ohio State Dental Society.*

Coming from the medical into the dental profession, I fancied that dentists had paid less attention to chemistry than to other sciences collateral with Dental Surgery. This fancy had much

A a B C D E b F G
30 40 50 60 70 80 90 100 110 120 130 110 150
1
2
3
4
5
6
7
8
9
10
11
12
13
14
15
16

### EXPLANATION OF SPECTRA.

1.—The spectrum of volatilized sodium.
2.—The two bands of oxidized blood; the two blood bands.
3.—Stokes' reduction band; band of reduced or de-oxidized blood.
4.—The two bands of oxidized blood, with the band in red, peculiar to old blood.
5.—The band in red peculiar to old blood, in a concentrated solution.
6.—Acid hæmatine, four banded.
7.—Acid hæmatine, in alcohol, four banded.
8.—Hæmatine, alkaline.
9.—Hæmatine, reduced.
10.—The two blood bands and the band of sulphuretted hydrogen in the red part of the spectrum.
11.—Nitrous-oxide bands.
12.—Band in red, belonging to foregoing spectrum where hyponitric, acid forms in the blood.
13.—Cruentine, alkaline.
14.—Cruentine, reduced.
15.—Methæmoglobin band of Hoppe Seyler.
16.—Spectrum from the ash of human and animal remains acidulated with chloric acid.

## Figures and Tables

**Figure f1:**