# Erratum

**Published:** 1878-02

**Authors:** 


### ERRATUM.

In consequence of various irregularities in the office where the January number of the REGISTER was printed, quite a number of annoying errors found place in the first article of that number, the chief of which we will here correct.

On page five, seventh line from top, between the words "space" and "by," read, in a wave like motion.

On page six, tenth line from top, place b between the letters "F" and "G."

On page eight, fifth line from top, read Schellin for "Schollin."

On page nine, line fourteen from top, read In, instead of "For," first word of line.

On page twelve, third line of fourth paragraph, for "globulin, read globin."

On page fifteen, eighth line from top, between the words "as" and "all," introduce the word nearly.

In ninth line, same page, between the words "changes" and "we," introduce the words, they produce.

On same page, seventh line from bottom, for "chemossys," read chemolysis.

On page sixteen, twelfth line from top, instead of the word "for," read far more.

On same page, in the first line of third paragraph, for "Young," read Zunz, and in the next line, for "Julenburg," read Eulenberg.

On page seventeen, in third line of fifth paragraph, for "blood," read broad,

Page eighteen, first line at top, after the word "hyponitric," read acid.

Same page, fourth paragraph, first line, instead of "by entering, read enters.

Page twenty, second line from bottom, omit "of the blood," and in same line read its, instead of "their."

On page twenty-one, seventh line from top, for "organize," read ozonize.

Same page, third paragraph, fourth line, the word mass should follow "gelatinous."

On page twenty-two, first line at top, read ozonize for "organize."

On page twenty-three, ninth line from bottom, substitute the word either for "it."

